# Ultra-low-dose computed tomography for torsion measurements of the lower extremities in children and adolescents

**DOI:** 10.1186/s13244-022-01257-w

**Published:** 2022-07-15

**Authors:** Stephan Waelti, Tim Fischer, Jennifer Griessinger, Johannes Cip, Tobias Johannes Dietrich, Michael Ditchfield, Thomas Allmendinger, Michael Messerli, Stefan Markart

**Affiliations:** 1 Department of Radiology and Nuclear Medicine, Children’s Hospital of Eastern Switzerland, Claudiusstrasse 6, 9006 St. Gallen, Switzerland; 2 Department of Radiology and Nuclear Medicine, Cantonal Hospital St. Gallen, St. Gallen, Switzerland; 3 Radiation Protection and Medical Physics, Cantonal Hospital Aarau, Aarau, Switzerland; 4 Department of Orthopedic Surgery, Children’s Hospital of Eastern Switzerland, St. Gallen, Switzerland; 5 Department of Diagnostic Imaging, Monash Children’s Hospital, Clayton, Australia; 6 Diagnostic Imaging, Computed Tomography, Siemens Healthcare, Forchheim, Germany; 7 Department of Nuclear Medicine, University Hospital Zurich, Zurich, Switzerland

**Keywords:** Femoral torsion, Tibial torsion, Children, Ultra-low-dose CT, Radiation dose

## Abstract

**Background:**

Quantifying femoral and tibial torsion is crucial in the preoperative planning for derotation surgery in children and adolescents. The use of an ultra-low-dose computed tomography (CT) protocol might be possible for modern CT scanners and suitable for reliable torsion measurements even though the bones are not completely ossified.

**Methods:**

This is a retrospective review of 77 children/adolescents (mean age 12.7 years) who underwent a lower extremity CT for torsion measurements on a 64-slice scanner. A stepwise dose reduction (70%, 50%, 30% of the original dose) was simulated. Torsion measurements were performed on all image datasets, and image noise, interrater agreement and subjective image quality were evaluated. Effective radiation dose of each original scan was estimated. As proof of concept, 24 children were scanned with an ultra-low-dose protocol, adapted from the 30% dose simulation, and the intra-class correlation coefficient (ICC) was determined. Ethics approval and informed consent were given.

**Results:**

Torsion measurements at the simulated 30% dose level had equivalent interrater agreement compared to the 100% dose level (ICC ≥ 0.99 for all locations and dose levels). Image quality of almost all datasets was rated excellent, regardless of dose. The mean sum of the effective dose of the total torsion measurement was reduced by simulation from 0.460/0.490 mSv (boys/girls) at 100% dose to 0.138/0.147 mSv at 30%. The ICC of the proof-of-concept group was as good as that of the simulated 30% dose level.

**Conclusion:**

Pediatric torsion measurements of the lower extremities can be performed using an ultra-low-dose protocol without compromising diagnostic confidence.

## Key points

- Pediatric CT torsion measurements can be carried out using an ultra-low-dose protocol.
- Skeletal immaturity does not compromise diagnostic accuracy.
- Images for angle measurements should be reconstructed using a soft tissue kernel.

## Introduction

Torsional pathology of the lower extremities is a common reason for consultation in pediatric orthopedics [1, 2]. Femoral and tibial torsion refers to rotation between the proximal and distal parts of the femur and tibia, respectively, assessed on a transverse plane [3]. The normal values of femoral antetorsion change during growth up to a normal range in adults [1]. In most cases of torsion anomaly, the cause is not known [1]. Known causes of torsional anomalies are cerebral palsy, myelomeningocele, previous femoral fracture or hip dysplasia [4, 5]. Femoral retrotorsion is known as a risk factor for osteoarthritis, femoroacetabular impingement and slipped capital femoral epiphysis [6–8]. Excessive femoral retrotorsion predisposes to recurrent patellar dislocation [9–14]. Quantification of femoral and tibial torsion is crucial in order to achieve exact preoperative planning for lower limb rotation-correcting surgery in children and adolescents [13, 15–21]. Various imaging techniques are available for the assessment of femoral and tibial torsion, including computed tomography (CT) [13, 22–27], low-dose biplanar radiography [13, 28–32] and magnetic resonance imaging (MRI) [8, 33, 34]. CT is considered the gold standard, and despite the inherent radiation exposure, CT is often used for quantifying torsional anomalies due to its widespread and short-term availability, proven accuracy, short examination time and cost-effectiveness, compared to other methods such as MRI [1, 35]. The short examination time is particularly advantageous for disabled patients.

If the torsion measurement is carried out using CT, then, according to the ALARA (as low as reasonably achievable) principle, the dose should be kept as low as possible. This is especially true for children and adolescents, which are considered to be more radiosensitive [13, 32]. Therefore, it is crucial to minimize radiation dose without compromising the diagnostic accuracy of the torsion measurements.

On the other hand, the bony structures are incompletely ossified in children, which can make it difficult to define the cortical bone and thus make a reliable angle measurement difficult, especially at low doses.

The aim of this retrospective single-center study was to evaluate reliability, image quality and estimated effective radiation dose of simulated ultra-low-dose CT in children and adolescents who underwent a clinically indicated CT scan for torsion measurements of the lower extremities.

## Materials and methods

### Patients

Between August 2020 and March 2021, 129 children underwent a CT scan of the lower extremities for torsion measurements at our Children’s hospital. After applying the inclusion criteria (written consent to the study, CT raw data still available, no metal implants), 77 children were retrospectively included. The study was approved by the local ethics committee. Written informed consent was obtained, signed either by the patients themselves (above the age of 14) or their parents (below the age of 14).

### Original CT examination

The scans were performed on a single-source 64-slice scanner (SOMATOM Definition AS, Siemens Healthineers, Forchheim, Germany) using our standard protocol. The legs were stabilized using tape around the feet. Three short scans were obtained on each patient, of the hip (scan range from superior border of acetabulum to lesser trochanter), knee (upper edge of the patella to proximal tibial metaphysis) and ankle (tibiofibular syndesmosis to distal fibular tip). For the hip scan, tube voltage and tube current were chosen by the scanner using automated tube voltage selection (ATVS) and automated tube current modulation (ATCM). Scan parameters are given in Table 1. Total scan time was 3–5 s, and overall examination time (including patient’s positioning) was about 5 min. None of the patients needed sedation.

**Table 1:** Scan parameters of the standard (original) cohort and the proof-of-concept cohort

Protocol	Hip		Knee		Ankle	
	Standard	Proof of concept	Standard	Proof of concept	Standard	Proof of concept
Automated tube current modulation + automated tube voltage selection	On	On	Off	Off	Off	Off
Tube voltage (kV)	100 (selected by software)	100 (selected by software)	80	80	80	80
Tube current–time product (mAs)	50^(*)^/70 (ref. mAs^(**)^)	15^(*)^/21 (ref. mAs^(**)^)	35	10	25	8
Tube filter	Wedge2^(*)^/flat	Wedge2^(*)^/flat	Wedge2^(*)^/flat	Wedge2^(*)^/flat	Wedge2^(*)^/flat	Wedge2^(*)^/flat
Pitch	0.8	0.8	0.8	1.0	0.8	1.25
Rotation time (s)	0.5	0.5	1	0.5	1	0.5
Total collimation (mm)	38.4 (0.6 * 64)	38.4 (0.6 * 64)	38.4 (0.6 * 64)	38.4 (0.6 * 64)	38.4 (0.6 * 64)	38.4 (0.6 * 64)
CTDI vol [mGy] (SSDE/Child phantom)	1.9 ± 0.6^(*)^/3.3 ± 0.6 (mean SSDE)	0.8 ± 0.1^(*)^/1.4 ± 0.3 (mean SSDE)	1.14^(*)^/1.48	0.32^(*)^/0.42	0.82^(*)^/1.06	0.24^(*)^/0.34
CTDI vol reduction (%)	55^(*)^–58%	72%	68–71^(*)^ %			

^(^*^)^Age < 12 years

^(^**^)^Actual effective cube current chosen by automated tube current modulation

### Simulation of ultra-low-dose CT

Based on the raw data of the original scan (dose considered as 100%), dose reductions of 70%, 50% and 30% of the original dose were simulated using the prototype reconstruction system ReconCT (Version 14.2.0.40998, Siemens Healthineers). ReconCT enables adding noise to the raw data prior to the image reconstruction and thereby simulates a dose reduction [36, 37]. The simulation has a limited reliability at extremely low dose values due to nonlinear systematic electronic noise effects and signal-dependent filtering [38]. Therefore, the maximum dose reduction is limited to 30% of the original dose.

### Image reconstruction

**Fig. 1:**
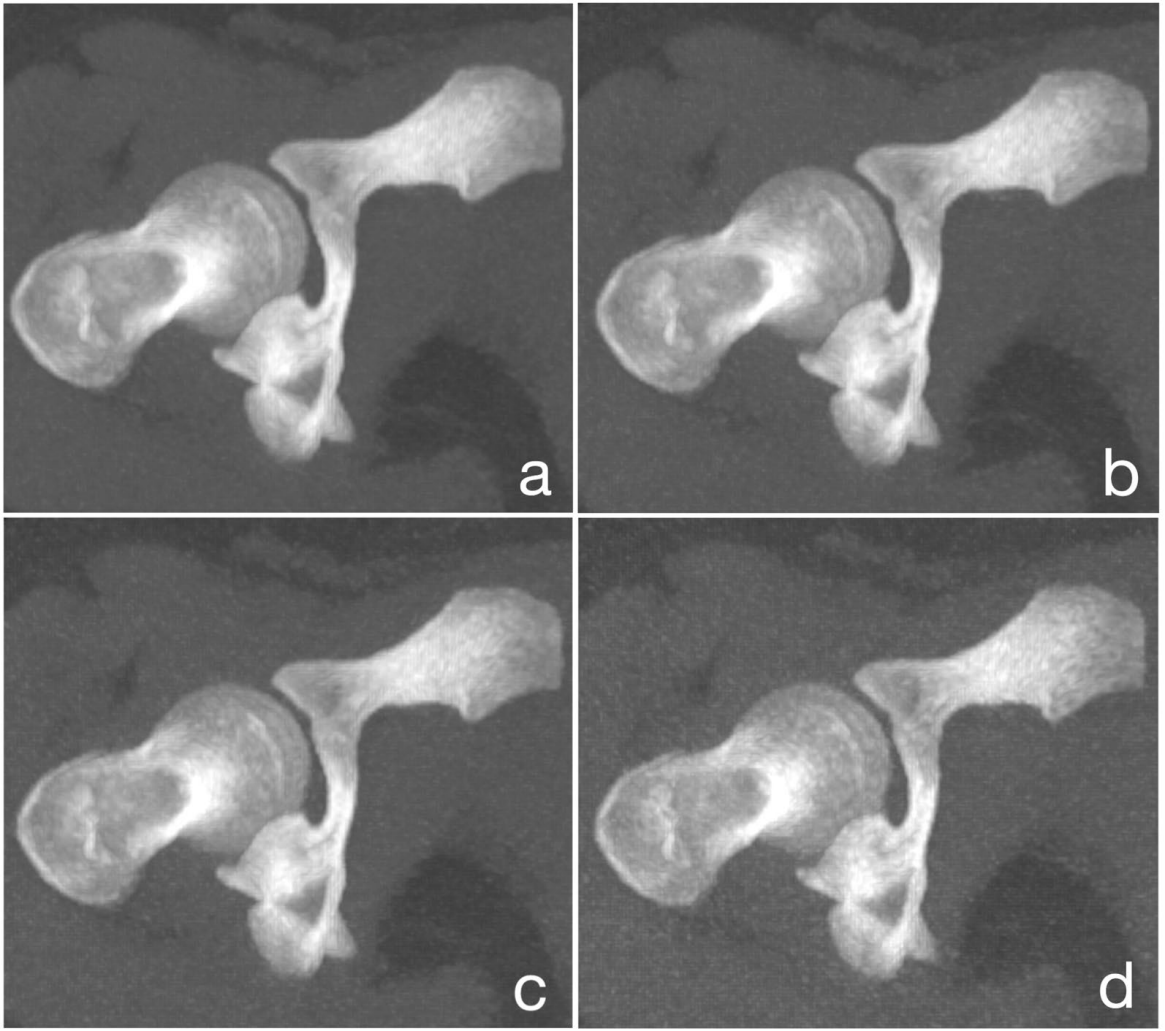
Sample images of the femoral neck reconstructed with a soft tissue kernel and using a bone window at all dose levels: 100% (**a**), 70% (**b**), 50% (**c**) and 30% (**d**)

Axial images were reconstructed in identical fashion at the individual dose levels (100%, 70%, 50%; 30%) using a model-based Advanced Modeled Iterative Reconstruction algorithm (ADMIRE, Siemens Healthineers) with a strength setting of 2 [39]. Separate reconstructions with a bone kernel Br59 and a soft tissue kernel Br37 were performed. Slice thickness was set to 5 mm for the hip and 3 mm for the knee and ankle reconstructions. Figure 1 shows sample images of every dose level reconstructed with a soft tissue kernel and using a bone window.

### Quantitative image quality analysis

On axial images, a circular 0.5 cm^2^ region of interest (ROI) was placed in the fatty tissue next to the rima ani immediately below the coccygeal tip (hip scan), between the biceps femoris and semimembranosus muscle tendons (knee scan), and in the Kager fat pad (ankle scan). ROIs were always placed by the same radiologist (S.M.) with 7 years of experience. Average attenuation values (in Hounsfield units, HU) and noise levels (SD of the mean Hounsfield unit) of every ROI and with both reconstruction kernels were noted.

### Subjective image quality analysis

Two pediatric radiologists with an overall experience of radiology of 7 (S.M.) and 11 years (S.W.) rated every dataset independently on axial bone window images with regard to perceptibility of cortical bone at the level of the below-mentioned angles, using a 4-point Likert scale: 1, excellent; 2, good; 3, fair; and 4, non-diagnostic.

### Angle measurements

Axial images in the bone window, which were reconstructed using a soft tissue kernel, were used for torsion measurements. Measurements were performed independently by the same two pediatric radiologists. Patients and dose levels were shown in a random order, and the readers were blinded to the results of the previous measurements and dose level. Angle measurements were performed using the angle measurement tool integrated in the picture archiving and communication system (PACS) used (Dedalus DeepUnity Diagnost 1.1.0.1, Germany).

**Fig. 2:**
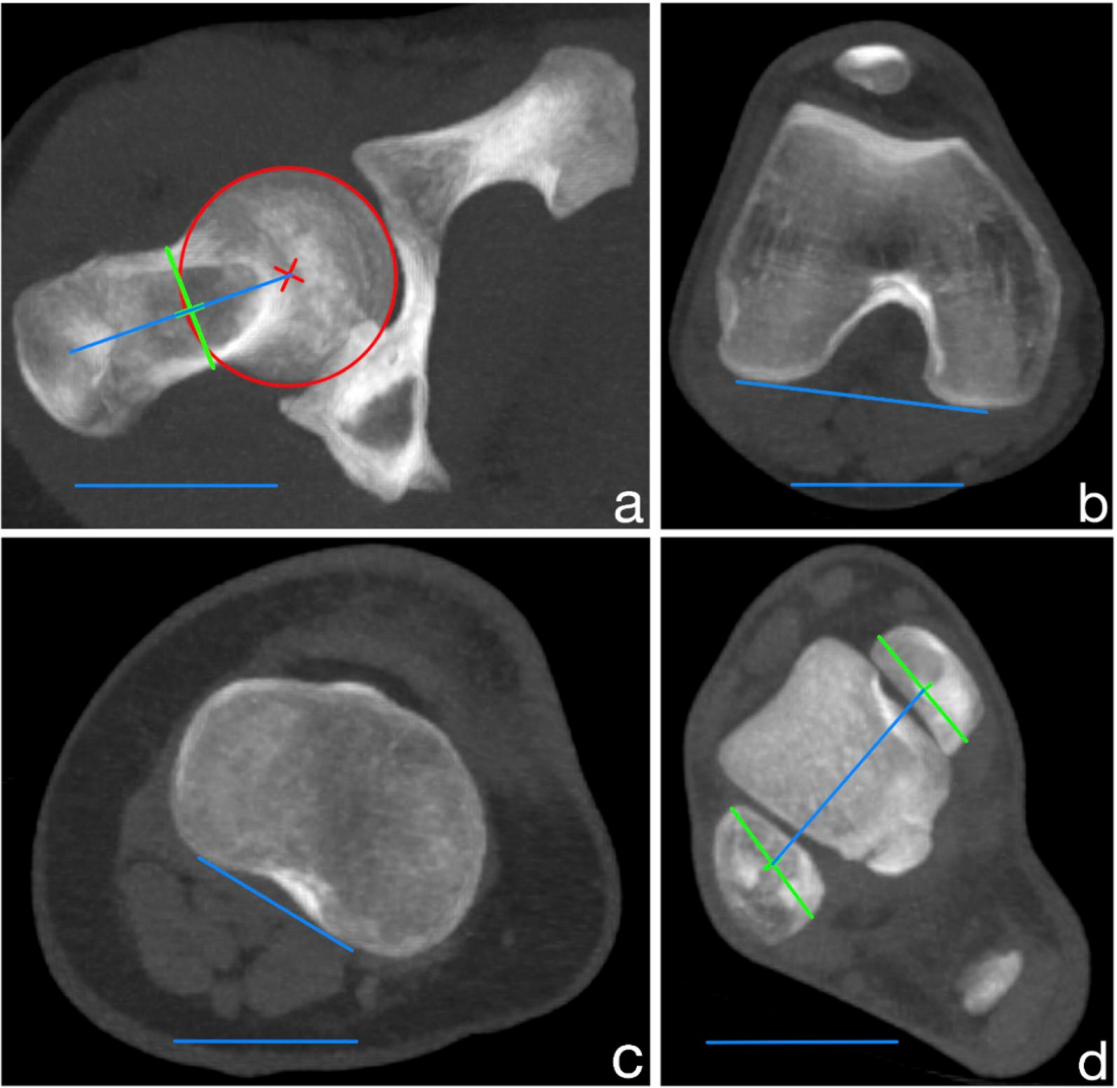
Angle measurements of the femoral neck (**a**), femoral condyle (**b**), tibial head (**c**) and ankle (**d**) using axial images in the bone window, which were reconstructed using a soft tissue kernel

*Femoral neck angle* was assessed using a technique modified from Yoshioka et al. [40]. The angle was drawn on transverse maximum intensity projection (MIP) images (30 mm) between a line from the center of the femoral head to the center of the femoral neck at its narrowest width and the horizontal line (Fig. 2a).

*Femoral condyle angle* was drawn on transverse MIP images (6 mm) between a tangent to the posterior border of the condyles at their maximum expansion from anterior to posterior and the horizontal line (Fig. 2b).

*Tibial head angle* was assessed using a modified technique described by Goutallier et al. [41]. The angle was drawn on transverse MIP images (6 mm) between a line along the posterior margin of the tibial plateau and the horizontal line (Fig. 2c).

*Intermalleolar angle* was drawn on transverse MIP images (6 mm) between a line through the midpoints of the maximal anteroposterior diameters of the medial and lateral malleolus and the horizontal line (Fig. 2d).

### Adapted (new) ultra-low-dose CT examination

After the statistical evaluation of the simulations showed that measurements are reliable at 30%, we adjusted the scan protocol of the same scanner to approach the 30% in a real-life scenario. For the hip scan, we reduced the reference mAs by 70%. For the knee and ankle scans, the tube current mAs values were reduced by 70%. Scan parameters are given in Table 1. This study extension was also approved by the local ethics committee, and written informed consent was obtained, signed either by the patients themselves (above the age of 14) or their parents (below the age of 14).

### Estimation of the effective radiation dose

Radiation dose was calculated using the software CT-Expo v2.3 [42]. As all CTDIvol and dose length product (DLP) values from the scanner were calibrated on the adult body phantom (32 cm diameter) for the estimation of the hip scans, size-specific dose estimates (SSDE) correction was performed for every patient according to the American Association of Physicists in Medicine (AAPM) report No. 204 [43]. Gender-specific estimation of the effective dose based on the child phantom (7 years/115 cm size/22 kg weight) was performed, transferring the scan region and length of the imaging data to the phantom for every patient. For the knee and ankle scans in the original cohort, the CTDIvol and DLP of the scanner were corrected for the child body phantom (16 cm diameter) and the estimation in CT-Expo was performed gender specifically on the child phantom mentioned above for 11 (female) and 12 (male) patients. The DLP (corrected) vs. the estimated effective dose was fitted for these 11/12 patients, and conversion factors (effective dose per DLP) were calculated gender specifically for the ankle and knee regions. Using these conversion factors, the estimated effective dose of the remaining patients was calculated. For the proof-of-concept cohort (*n* = 24), all scans were estimated as described above using CT-Expo v2.3.

### Statistical analysis

Image noise was compared between the different radiation doses with a Spearman’s rho correlation. The association of age and dose with diagnostic confidence was assessed with Wilcoxon rank sum tests and Fisher’s exact tests, respectively. The intra-class correlation coefficient (ICC) was computed to assess the agreement between two radiologists rating the torsion angles. The ICC2 (two-way random-effects model for single rating) was computed. ICC is interpreted as poor for values below 0.50, as moderate for values between 0.50 and 0.75, as good for values between 0.75 and 0.90 and as excellent for values above 0.90 [44]. Moreover, mean differences with limits of agreement (corresponding to 1.96 times the standard deviation) were computed and visualized with Bland–Altman plots. Bland–Altman plots were plotted to show interrater agreement. All analyses were performed in the R programming language (version 4.0.2). The package “tableone” was used to compute descriptive statistics, the package “ggplot2” was used to plot the Bland–Altman plots, and the package “psych” was used to compute the ICC.

## Results

### Patients

The cohort scanned with the standard protocol consisted of a total of 77 children, 28 girls (36.4%) and 49 boys (63.6%). The mean age for both sexes combined was 12.7 years (9.0–17.0 years), for girls 13.0 years (10.0–17.0 years) and for boys 12.5 years (9.0–17.0 years). The mean anteroposterior pelvic diameter measured 18.0 cm (range 13.3–28.0 cm) for both genders combined, 17.6 cm (14.1–22.1 cm) for girls and 18.2 cm (13.3–28.0 cm) for boys. The mean transverse pelvic diameter measured 33.1 cm (range 22.7–42.0 cm) for both genders combined, 33.4 cm (25.3–41.2 cm) for girls and 32.9 cm (22.7–42.0 cm) for boys.

### Objective image quality

For all locations and doses, mean image noise was higher for the bone kernel than for the soft tissue kernel. Image noise was highest for the fat tissue next to the rima ani and lowest for the Kager fat pad. Image noise increased with decreasing dose levels (*p* < 0.001 for all locations and both kernels). For the 100% dose level, mean image noise SD (soft tissue kernel) for the hip, knee and ankle scan was 37.6, 34.0 and 24.6 HU, respectively, and increased by 70–78% for the 30% dose level.

### Subjective image quality

Diagnostic confidence was rated as Likert scores 1 (excellent) and 2 (good) for all studies, none receiving a Likert score 3 (fair) or 4 (non-diagnostic). The vast majority of all scans were ranked as excellent (Likert score 1) by both readers (98% and 99%, respectively). Likert score 2 was given in 2% (reader 1) and 1% (reader 2) of all scans. Furthermore, Likert score 2 was mainly given for the tibial head by both readers (in 5% and 6%, respectively, of all tibial head scans). Two scans of the femoral neck with the 30% dose level and one scan of the ankle with the 30% dose level were ranked as only good by reader 2 (Likert scores 2). Age did not seem to be associated with diagnostic confidence.

### Angle measurements

Overall, the mean difference between reader 1 and 2 did not exceed 1 degree and the limits of agreement ranged between − 2.1° and 3.6°. Moreover, the 30% radiation dose level did not result in reduced interrater agreement. This was confirmed by a very high intra-class correlation coefficient (ICC) of at least 0.99 for all locations and dose levels (Table 2).

**Table 2:** Interrater agreement: intra-class correlation coefficient (ICC) and mean difference (degree) between readers 1 and 2 with corresponding limits of agreement

	Femoral neck	Femoral condyle	Tibial head	Ankle
Mean difference (limits of agreement), 100%	0.6 (− 2.1 to 3.3)	− 0.0 (− 1.1 to 1.0)	0.1 (− 1.9 to 2.0)	− 0.4 (− 1.9 to 1.2)
Mean difference (limits of agreement), 70%	0.7 (− 1.8 to 3.1)	− 0.1 (− 1.1 to 0.9)	0.1 (− 1.8 to 2.0)	− 0.3 (− 1.7 to 1.1)
Mean difference (limits of agreement), 50%	0.9 (− 1.8 to 3.5)	− 0.1 (− 1.2 to 1.0)	0.2 (− 1.8 to 2.1)	− 0.3 (− 1.8 to 1.3)
Mean difference (limits of agreement), 30%	1.0 (− 1.7 to 3.6)	− 0.1 (− 1.2 to 1.0)	0.2 (− 1.9 to 2.4)	− 0.2 (− 1.5 to 1.1)
ICC (95% CI), 100%	0.991 (0.985–0.994)	0.999 (0.998–0.999)	0.994 (0.992–0.996)	0.997 (0.994–0.998)
ICC (95% CI), 70%	0.992 (0.985–0.995)	0.999 (0.998–0.999)	0.994 (0.993–0.996)	0.997 (0.996–0.998)
ICC (95% CI), 50%	0.99 (0.977–0.995)	0.999 (0.998–0.999)	0.994 (0.992–0.995)	0.997 (0.996–0.998)
ICC (95% CI), 30%	0.989 (0.971–0.995)	0.998 (0.998–0.999)	0.993 (0.99–0.994)	0.998 (0.997–0.999)

**Fig. 3:**
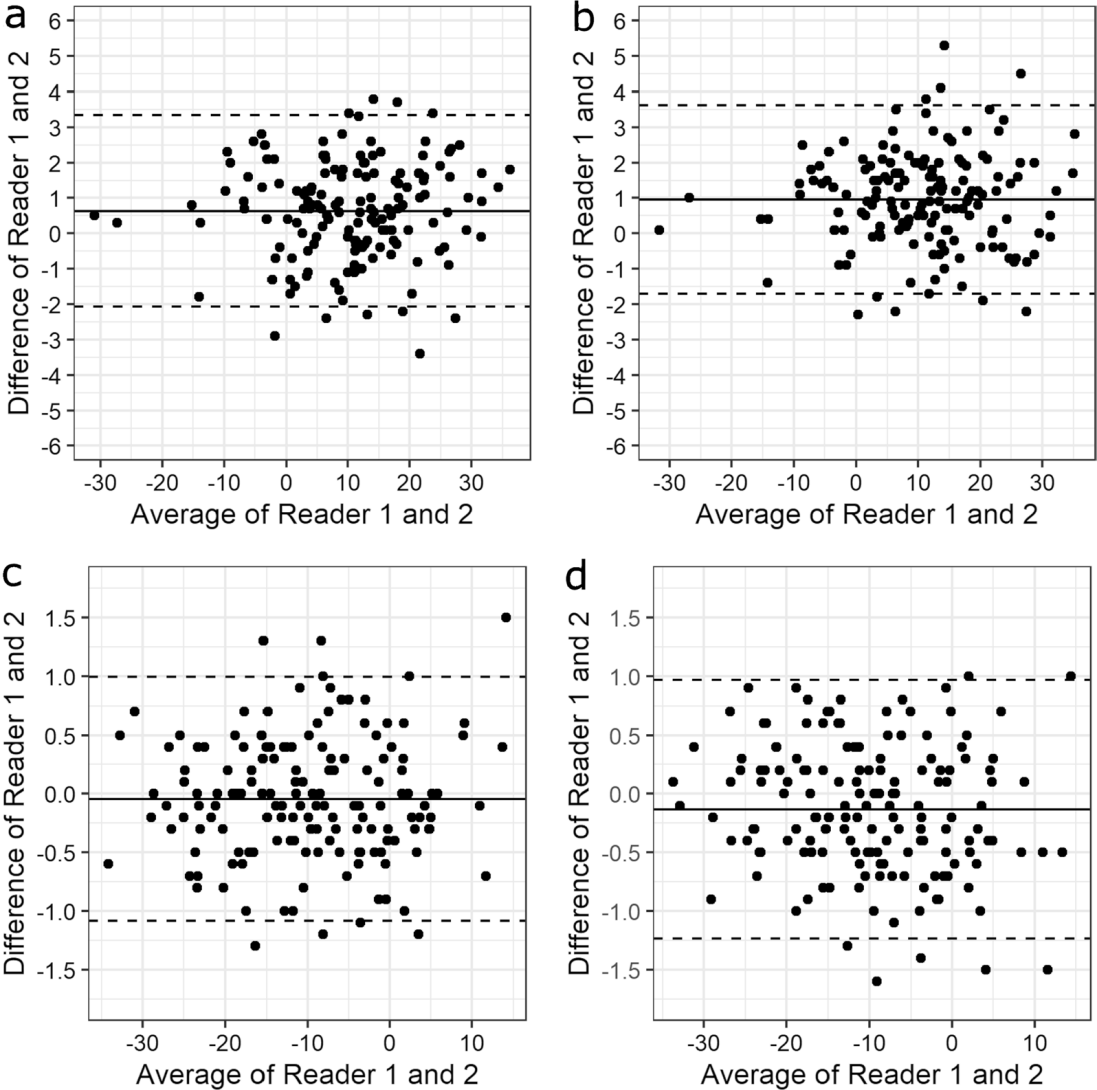
Bland–Altman plots for the femoral neck with dose levels 100% (**a**) and 30% (**b**) and for the femoral condyle with dose levels 100% (**c**) and 30% (**d**). The solid line illustrates the mean difference and the dashed lines indicate average difference ± 1.96 * standard deviation of the difference

**Fig. 4:**
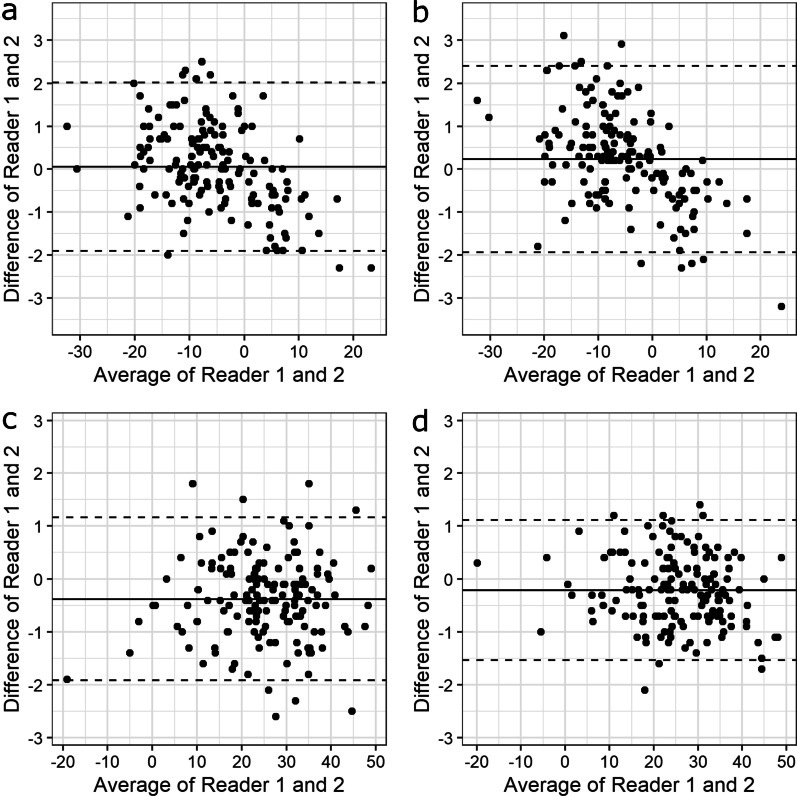
Bland–Altman plots for the tibial head with dose levels 100% (**a**) and 30% (**b**) and for the ankle with dose levels 100% (**c**) and 30% (**d**). The solid line illustrates the mean difference and the dashed lines indicate average difference ± 1.96 * standard deviation of the difference

With a maximum absolute mean difference of 1.0°, the estimated bias was small for all locations and dose levels. The limits of agreement were smallest for femoral condyle and highest for femoral neck. However, they were similar for 100% and 30% dose levels for all locations (Figs. 3, 4).

### Proof of concept

A total of 24 patients (female: 16; male: 8; mean age: 13.0 years) were scanned using an ultra-low-dose protocol. The interrater agreement was similarly good compared to the 100% dose level in the main patient cohort. Thus, the mean difference did not exceed 0.3 degree and the limits of agreement ranged between − 1.9° and 2.4°. The ICC was high with values between 0.996 and 0.999 (Table 3). The Bland–Altman plots (not shown) indicated similar results to the simulated 30% dose level data. Therefore, the absolute mean differences were between 0.0° (femoral condyle) and 0.3° (femoral neck) with limits of agreement that were comparable to the simulated 30% dose level data.

**Table 3:** Intra-class correlation coefficient (ICC) and mean difference between raters 1 and 2 with corresponding limits of agreement (proof-of-concept group)

	Femoral neck	Femoral condyle	Tibial head	Ankle
Mean difference (limits of agreement), 30%	0.3 (− 1.9 to 2.4)	0.0 (− 0.9 to 1.0)	0.2 (− 1.6 to 2.0)	− 0.1 (− 1.5 to 1.4)
ICC (95% CI), 30%	0.996 (0.994–0.998)	0.999 (0.998–0.999)	0.996 (0.993–0.997)	0.998 (0.997–0.999)

### Dose estimation

For the original protocol, the summed effective doses ranged from 0.20 mSv (10 years old, 19 cm eff. diameter) to 0.96 mSv (14 years old, 28 cm eff. diameter) for boys and from 0.19 mSv (10 years old, 19 cm eff. diameter) to 0.93 mSv (13 years old, 21 cm eff. diameter) for girls. This results in a dose range for the simulated 30% dose level of 0.06–0.29 mSv for boys and 0.06–0.28 mSv for girls. Table 4 summarizes the mean values of the effective dose for the original scan (100%) and the simulated dose reductions (70%, 50%; 30%).

**Table 4:** Mean effective dose for the original scan (100%) and the simulated dose reductions (70%, 50%, 30%) for boys and girls, the respective scan region (hip, knee, ankle) and the sum of the effective dose (mean)

Dose	Mean effective dose hip boys (mSv)	Mean effective dose hip girls (mSv)	Mean effective dose knee boys (mSv)	Mean effective dose knee girls (mSv)	Mean effective dose ankle boys (mSv)	Mean effective dose ankle girls (mSv)	Sum of effective dose boys (mean) (mSv)	Sum of effective dose girls (mean) (mSv)
100%SD	0.420 ± 0.172	0.450 ± 0.173	0.030 ± 0.006	0.030 ± 0.005	0.010 ± 0.002	0.010 ± 0.003	0.460 ± 0.177	0.490 ± 0.178
70%	0.294	0.315	0.021	0.021	0.007	0.007	0.322	0.343
50%	0.210	0.225	0.015	0.015	0.005	0.005	0.230	0.245
30%	0.126	0.135	0.009	0.009	0.003	0.003	0.138	0.147

SD, standard deviation

**Fig. 5:**
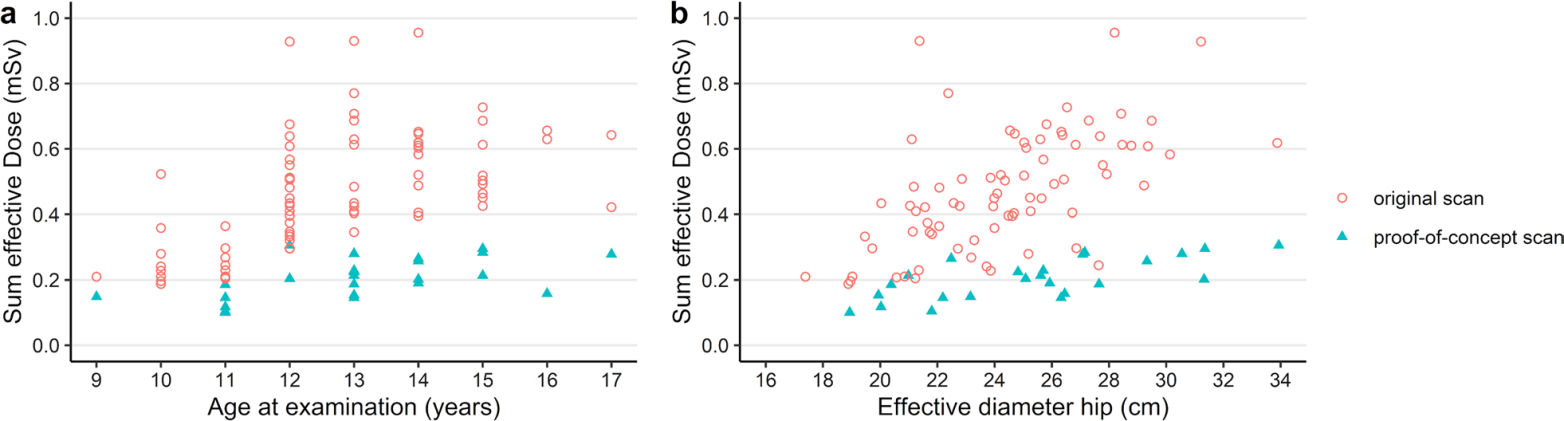
Summed effective dose for the original protocol and the proof-of-concept protocol. The distribution of age (**a**) and hip diameter (**b**) are similar in both groups. Hip diameter is required for size-specific dose estimation

For the proof-of-concept group, summed effective doses ranged from 0.10 mSv (11 years old, 19 cm eff. diameter) to 0.28 mSv (14 years old, 26 cm eff. diameter) for male and 0.10 mSv (11 years old, 22 cm eff. diameter) to 0.30 mSv (12 years old, 34 cm eff. diameter) for female patients, showing dose reduction of 45–70%, with a mean dose reduction of 60% (male) and 56% (female), respectively. Figure 5a, b demonstrates the dose reduction plotted against the age and the effective diameter of the patients’ hip.

For the proof-of-concept group, the mean CTDIvol values for the hip (SSDE corrected) as well as the fixed CTDIvol values for the knee and ankle (child phantom corrected) revealed a dose reduction of 55–58% for the hip, 72% for the knee and 68–71% for the ankle scans, respectively. On this basis, the corrected CTDIvol values revealed a dose level of 28–45% in the adapted ultra-low-dose protocols compared to the original scan protocols.

## Discussion

In this study, we have attempted to push the limits of the ALARA principle in the assessment of torsion measurements of the lower extremities using CT in children and adolescents. Despite the skeletal immaturity, the bony landmarks are still identified and CT torsion measurements are reliable even in the ultra-low-dose range.

In children and adolescents, torsion measurement without or with a small amount of ionizing radiation, i.e., MR or low-dose biplanar radiography, should be preferred. However, MR is less available, takes more time, costs more and has limitations in patients with metal implants [13, 32–34, 45]. It is also prone to motion artifacts, which is a particular problem with disabled children. However, because exact preoperative radiological determination of torsional deformities is crucial for orthopedic surgeons in order to achieve an accurate postoperative result, the radiological examination must be as precise as possible. Low-dose biplanar radiography is available in only a few hospitals. Therefore, referring physicians continue to request CT scans because it is more readily available, is less susceptible to motion artifacts and takes a very short time so that sedation is usually not necessary and the patient can then return to the clinic immediately to discuss the results and management.

Due to the inaccuracy of the simulations below 30%, we did not simulate a dose reduction below 30%. However, the study by Keller et al. [35] in adult patients suggests that a further dose reduction might be possible, but their mean initial dose was approximately 8 times greater than our mean initial dose (~ 4 mSv vs. ~ 0.5 mSv). Based on the scan parameters, the dose of our original standard protocol is also significantly lower compared to the pediatric torsion CT study by Rosskopf et al. [13], which was performed with an older 64-slice CT system from a different vendor. Further dose reduction will be possible in newer scanners by using tin filters for spectral shaping [46].

We consider the variance of the measurements at the level of the femoral neck to be mainly due to the measurement method and not to the lower dose. If the reader does not select the exact same slices, this can lead to measurement differences. The variance of the measurements at the level of the tibial head is mainly due to the fact that the tibial epiphysis in children has a more ovoid configuration than in adults [13].

Despite the substantial increase in image noise, even the lowest simulated dose (30%) shows no significant difference in the accuracy of the angle measurements compared to the original dose (100%). The proof-of-concept group has confirmed that this is also true in clinical practice and we recommend that these scans should be performed at this or even lower dose in the future. Noise is significantly lower in images reconstructed with a soft tissue kernel than in images reconstructed with a bone kernel. Therefore, we recommend to perform angle measurements on images reconstructed with a soft tissue kernel.

In the proof-of-concept group, CTDIvol reduction to 30% of baseline was possible at the level of the knee and ankle. At the level of the pelvis, the reduction of reference mAs to 30% of baseline was insufficient to achieve a 70% CTDIvol reduction. Instead, a 55–58% reduction in CTDIvol was achieved (Table 1). Thus, at the pelvis, further adjustments of the parameters are necessary.

Model-based iterative reconstruction algorithms may be compromised at low doses due to the higher noise. In the future, deep-learning-based image reconstruction techniques will be increasingly applied, allowing fast, low-noise reconstruction with high spatial resolution despite the low dose. Deep-learning-based algorithms are trained with high-quality, advanced model-based, iterative reconstruction images with which it learns to transform poor input data into low-noise, sharp and clear images [47].

This study has some limitations. Firstly, in the current study due to technical reasons and in close collaboration with the manufacturer we were only able to reduce the dose level to 30%, which may mean that the true potential of dose reduction for this clinical task is indeed underestimated. We strongly encourage further clinical studies with the aim of further dose reduction in pediatric patients. Secondly, the conclusion and the dose recommendations derived from this work are in principle only valid for this specific iterative reconstruction algorithm and thus in this case for ADMIRE. The respective reconstructions are usually tailored to the individual scanners and generalization and transfer to iterative reconstructions, even if “model-based,” of other manufacturers is usually not given. However, we strongly encourage users of scanners from other manufacturers to also attempt dose reduction for torsion CT examinations of children and adolescents. Thirdly, we excluded patients with metal implants (e.g., epiphysiodesis implants). Hardening artifact from metallic objects may make ultra-low-dose protocols impossible or interfere with the automatic dose correction of the scanner. Fourthly, we used chronological age instead of skeletal age. Fifthly, none of our patients were younger than 9 years old. However, the vast majority of derotational osteotomies are performed in older children and adolescents [13, 48].

## Conclusion

CT torsion measurements of the lower extremities in children and adolescents can be carried out using an ultra-low-dose protocol. Even in children with skeletal immaturity, torsion measurements can be performed with such a protocol without compromising diagnostic accuracy. An ultra-low-dose protocol can be carried out with a modern CT system. Images for angle measurements should be reconstructed using a soft tissue kernel.

## Data Availability

The datasets used and/or analyzed during the current study are available from the corresponding author on reasonable request.

## Abbreviations

AAPM: American Association of Physicists in Medicine
ADMIRE: Advanced Modeled Iterative Reconstruction
ALARA: As low as reasonably achievable
ATCM: Automated tube current modulation
ATVS: Automated tube voltage selection
CT: Computed tomography
CTDI: Computed tomography dose index
DLP: Dose length product
HU: Hounsfield unit
ICC: Intra-class correlation coefficient
MIP: Maximum intensity projection (MIP)
MRI: Magnetic resonance imaging
PACS: Picture archiving and communication system
ROI: Region of interest
SD: Standard deviation
SSDE: Size-specific dose estimates

## References

[1] Grisch D, Dreher T (2019). Torsion and torsional development of the lower extremities. Orthopade.

[2] Silva MS, Fernandes AR, Cardoso FN, Longo CH, Aihara AY (2019). Radiography, CT, and MRI of hip and lower limb disorders in children and adolescents. Radiographics.

[3] Kaiser P (2016). Significant differences in femoral torsion values depending on the CT measurement technique. Arch Orthop Trauma Surg.

[4] Zeckey C (2017). Femoral malrotation after surgical treatment of femoral shaft fractures in children: a retrospective CT-based analysis. Eur J Orthop Surg Traumatol.

[5] Johnson DC, Damiano DL, Abel MF (1997). The evolution of gait in childhood and adolescent cerebral palsy. J Pediatr Orthop.

[6] Tonnis D, Heinecke A (1999). Acetabular and femoral anteversion: relationship with osteoarthritis of the hip. J Bone Joint Surg Am.

[7] Gelberman RH, Cohen MS, Shaw BA, Kasser JR, Griffin PP, Wilkinson RH (1986). The association of femoral retroversion with slipped capital femoral epiphysis. J Bone Joint Surg Am.

[8] Botser IB, Ozoude GC, Martin DE, Siddiqi AJ, Kuppuswami S, Domb BG (2012). Femoral anteversion in the hip: comparison of measurement by computed tomography, magnetic resonance imaging, and physical examination. Arthroscopy.

[9] Dickschas J, Harrer J, Reuter B, Schwitulla J, Strecker W (2015). Torsional osteotomies of the femur. J Orthop Res.

[10] Fithian DC (2004). Epidemiology and natural history of acute patellar dislocation. Am J Sports Med.

[11] Hinterwimmer S, Rosenstiel N, Lenich A, Waldt S, Imhoff AB (2012). Femoral osteotomy for patellofemoral instability. Unfallchirurg.

[12] Post WR, Teitge R, Amis A (2002). Patellofemoral malalignment: looking beyond the viewbox. Clin Sports Med.

[13] Rosskopf AB, Ramseier LE, Sutter R, Pfirrmann CW, Buck FM (2014). Femoral and tibial torsion measurement in children and adolescents: comparison of 3D models based on low-dose biplanar radiography and low-dose CT. AJR Am J Roentgenol.

[14] Dejour D, Le Coultre B (2007). Osteotomies in patello-femoral instabilities. Sports Med Arthrosc Rev.

[15] Aird JJ, Hogg A, Rollinson P (2009). Femoral torsion in patients with Blount's disease: a previously unrecognised component. J Bone Joint Surg Br.

[16] Huber H, Haefeli M, Dierauer S, Ramseier LE (2009). Treatment of reduced femoral antetorsion by subtrochanteric rotational osteotomy. Acta Orthop Belg.

[17] Lee SH, Chung CY, Park MS, Choi IH, Cho TJ (2009). Tibial torsion in cerebral palsy: validity and reliability of measurement. Clin Orthop Relat Res.

[18] Noyes FR, Goebel SX, West J (2005). Opening wedge tibial osteotomy: the 3-triangle method to correct axial alignment and tibial slope. Am J Sports Med.

[19] Song HR (2006). Rotational profile of the lower extremity in achondroplasia: computed tomographic examination of 25 patients. Skeletal Radiol.

[20] Abadie P, Galaud B, Michaut M, Fallet L, Boisrenoult P, Beaufils P (2009). Distal femur rotational alignment and patellar subluxation: a CT scan in vivo assessment. Orthop Traumatol Surg Res.

[21] Karaman O, Ayhan E, Kesmezacar H, Seker A, Unlu MC, Aydingoz O (2014). Rotational malalignment after closed intramedullary nailing of femoral shaft fractures and its influence on daily life. Eur J Orthop Surg Traumatol.

[22] Hernandez RJ, Tachdjian MO, Poznanski AK, Dias LS (1981). CT determination of femoral torsion. AJR Am J Roentgenol.

[23] Jarrett DY, Oliveira AM, Zou KH, Snyder BD, Kleinman PK (2010). Axial oblique CT to assess femoral anteversion. AJR Am J Roentgenol.

[24] Murphy SB, Simon SR, Kijewski PK, Wilkinson RH, Griscom NT (1987). Femoral anteversion. J Bone Joint Surg Am.

[25] Waidelich HA, Strecker W, Schneider E (1992). Computed tomographic torsion-angle and length measurement of the lower extremity: the methods, normal values and radiation load. Rofo.

[26] Liodakis E (2012). Reliability of the assessment of lower limb torsion using computed tomography: analysis of five different techniques. Skeletal Radiol.

[27] Shin SY (2011). The availability of radiological measurement of tibial torsion: three-dimensional computed tomography reconstruction. Ann Rehabil Med.

[28] Meyrignac O (2015). Low-dose biplanar radiography can be used in children and adolescents to accurately assess femoral and tibial torsion and greatly reduce irradiation. Eur Radiol.

[29] Buck FM, Guggenberger R, Koch PP, Pfirrmann CW (2012). Femoral and tibial torsion measurements with 3D models based on low-dose biplanar radiographs in comparison with standard CT measurements. AJR Am J Roentgenol.

[30] Gheno R, Nectoux E, Herbaux B (2012). Three-dimensional measurements of the lower extremity in children and adolescents using a low-dose biplanar X-ray device. Eur Radiol.

[31] Thelen P, Delin C, Folinais D, Radier C (2012). Evaluation of a new low-dose biplanar system to assess lower-limb alignment in 3D: a phantom study. Skeletal Radiol.

[32] Rosskopf AB, Buck FM, Pfirrmann CW, Ramseier LE (2017). Femoral and tibial torsion measurements in children and adolescents: comparison of MRI and 3D models based on low-dose biplanar radiographs. Skeletal Radiol.

[33] Schneider B, Laubenberger J, Jemlich S, Groene K, Weber HM, Langer M (1997). Measurement of femoral antetorsion and tibial torsion by magnetic resonance imaging. Br J Radiol.

[34] Tomczak RJ, Guenther KP, Rieber A, Mergo P, Ros PR, Brambs HJ (1997). MR imaging measurement of the femoral antetorsional angle as a new technique: comparison with CT in children and adults. AJR Am J Roentgenol.

[35] Keller G, Afat S, Ahrend MD, Springer F (2021). Diagnostic accuracy of ultra-low-dose CT for torsion measurement of the lower limb. Eur Radiol.

[36] Ellmann S (2016). Dose reduction potential of iterative reconstruction algorithms in neck CTA-a simulation study. Dentomaxillofac Radiol.

[37] Kramer M (2015). Computed tomography angiography of carotid arteries and vertebrobasilar system: a simulation study for radiation dose reduction. Medicine (Baltimore).

[38] Duan X (2013). Electronic noise in CT detectors: impact on image noise and artifacts. AJR Am J Roentgenol.

[39] Newell JD (2015). Very low-dose (0.15 mGy) chest CT protocols using the COPDGene 2 test object and a third-generation dual-source CT scanner with corresponding third-generation iterative reconstruction software. Invest Radiol.

[40] Yoshioka Y, Cooke TD (1987). Femoral anteversion: assessment based on function axes. J Orthop Res.

[41] Goutallier D, Van Driessche S, Manicom O, Ali ES, Bernageau J, Radier C (2006). Influence of lower-limb torsion on long-term outcomes of tibial valgus osteotomy for medial compartment knee osteoarthritis. J Bone Joint Surg Am.

[42] Stamm G, Nagel HD (2002). CT-expo–a novel program for dose evaluation in CT. Rofo.

[43] AAPM (2011) A.-A.A.o.P.i.M., Report 204: size-specific dose estimates (SSDE) in pediatric and adult body CT examinations. https://aapm.org/pubs/reports/RPT_204.pdf 10.1118/1.4725757 PMC3412437 28516563

[44] Koo TK, Li MY (2016). A guideline of selecting and reporting intraclass correlation coefficients for reliability research. J Chiropr Med.

[45] Koenig JK, Pring ME, Dwek JR (2012). MR evaluation of femoral neck version and tibial torsion. Pediatr Radiol.

[46] Messerli-Odermatt O (2020). Chest X-ray dose equivalent low-dose CT with tin filtration: potential role for the assessment of pectus excavatum. Acad Radiol.

[47] Boedeker K (2017) Aquilion precision ultra-high resolution CT: quantifying diagnostic image quality, Canon Medical Systems Corporation

[48] Gordon JE, Pappademos PC, Schoenecker PL, Dobbs MB, Luhmann SJ (2005). Diaphyseal derotational osteotomy with intramedullary fixation for correction of excessive femoral anteversion in children. J Pediatr Orthop.

